# Gray literature in systematic reviews on population health in the Middle East and North Africa: protocol of an overview of systematic reviews and evidence mapping

**DOI:** 10.1186/s13643-018-0751-4

**Published:** 2018-07-18

**Authors:** Karima Chaabna, Sohaila Cheema, Amit Abraham, Hekmat Alrouh, Ravinder Mamtani, Javaid I. Sheikh

**Affiliations:** 1 Institute for Population Health, Weill Cornell Medicine—Qatar, P.O. Box 24144, Doha, Qatar; 2 Office of the Dean, Weill Cornell Medicine—Qatar, Doha, Qatar

**Keywords:** Gray literature, Population health, Public health, MENA, North Africa, Middle East, Arab countries, Middle East and North Africa, Overview of systematic reviews

## Abstract

**Background:**

Systematic review (SR) guidelines recommend extending literature search to gray literature in order to identify all available data related to the review topic. We aim to conduct an overview of SRs on population health in the Middle East and North Africa (MENA), to assess the methodology of these SRs, to produce an evidence map highlighting methodological gaps in SRs regarding gray literature searching, and to aid in developing future SRs by listing gray literature sources related to population health in MENA.

**Methods/design:**

We will conduct an overview of SRs based on the Cochrane Handbook for Systematic Reviews of Interventions. This overview will be reported following PRISMA 2009 guidelines. Using comprehensive search criteria, we will search the PubMed database to identify relevant SRs published since 2008. Our primary outcomes are gray literature sources and study-level quality in the gray literature. We will include MENA countries with Arabic, English, French, and/or Urdu as primary official languages and/or media of instruction in universities. Two reviewers will independently conduct a multilevel screening on Rayyan software. Extraction of relevant data will be done on Statistical Package for the Social Sciences (SPSS) software. The methodological quality of included SRs will be assessed using the Assessment of Multiple Systematic Reviews (AMSTAR) tool. Any disagreements will be resolved by discussion and consensus.

We will estimate the overall proportion of SRs that used gray literature as one of their data sources. Subgroup analyses will be conducted to identify characteristics of these gray literature sources. Chi-squared and *t* tests will be used to determine whether the differences between subgroups are statistically significant. Additionally, an evidence gap map will be constructed to highlight characteristics and quality of the gray literature used in SRs on population health in MENA and emphasize existing gaps in gray literature searching. We will also list gray literature sources identified in the included SRs stratified by country and research topic.

**Discussion:**

This overview will comprehensively assess the overall quality of the SRs on population health issues in MENA. Our findings will contribute to the improvement of population health research practices in MENA.

**Systematic review registration:**

The systematic review protocol was registered with the International Prospective Register of Systematic Reviews (PROSPERO) on 26 October 2018 (registration number CRD42017076736 (Syst Rev 2:4, 2013).

**Electronic supplementary material:**

The online version of this article (10.1186/s13643-018-0751-4) contains supplementary material, which is available to authorized users.

## Background

In healthcare, systematic reviews (SRs) and meta-analyses have become increasingly essential. Researchers, clinicians, and policymakers read them to keep up to date with their field and to make decisions [1, 2]. Thus, well-conducted SRs are important to implementing evidence-based medicine [3]. In 2008, the Institute of Medicine (IOM) recommended the development of methodological standards for SRs. Over the last decade, detailed materials guiding reviewers have been published; the original version of the Cochrane Handbook for Systematic Reviews of Interventions (version 5.0.1) was published in 2008 [4], the Preferred Reporting Items for Systematic Reviews and Meta-Analyses (PRISMA 2009) statement was published in 2009 [5], and IOM standards for SRs was published in 2011 [6]. These guidelines recommend extending literature searches to gray literature in order to identify all available data related to the review topic. The use of gray literature is important as it is likely to lead to a more complete view of available evidence [7]. Reviewers searching for gray literature on population health in the Middle East and North Africa (MENA) region might be challenged as the information may not be easy to search and retrieve because there were no central sources, which implies allocating considerable time and effort [7–9].

Non-English gray literature has been demonstrated to not affect results of reviews of intervention studies included in Cochrane Review Groups [10]. We will explore the use of gray literature by SRs of observational studies on population health in the MENA countries where English is not an official language. Our project objectives are to (i) conduct an overview of SRs (i.e., a systematic review of systematic reviews) on population health in MENA, (ii) assess the methodology of these SRs, (iii) produce an evidence gap map highlighting methodological gaps in SRs regarding gray literature searching, and (iv) aid in developing future SRs by listing gray literature sources related to population health in MENA.

## Methods

Our protocol has been registered on PROSPERO (PROSPERO registration number CRD42017076736 [11]) and is reported following the items outlined in Preferred Reporting Items for Systematic Reviews and Meta-Analyses for Protocols (PRISMA-P 2015) [12]. Completed PRISMA-P 2015 checklist [13] can be found in Additional file 1.

### Research questions to be addressed

The research questions we want to address are (i) what are the sources of gray literature used in the SRs on population health in MENA, (ii) what proportion of these SRs use gray literature, (iii) what are the characteristics of gray literature sources included in these SRs, (iv) are there differences between the SRs that use gray literature and those that do not, (v) are there differences between SRs using gray literature according to the type of gray literature, and (vi) what is the proportion of good quality studies from gray literature included in SRs on population health in MENA?

### Eligibility criteria

Our study is an overview of published systematic reviews. As such, we will include only SRs [4]. Narrative reviews will be excluded. We will consider a publication as being an SR if it was stated that the publication is an SR and a systematic literature search of at least one electronic database was conducted and described in the method section along with a description of eligibility criteria and study selection. Reviews not reporting a systematic methodology will be excluded.

The following eligibility criteria are designed in compliance with the population-intervention-comparator-outcome (PICO) model [14]. Populations in MENA are our populations of interest. We will use MENA region definition of The World Bank [15], World Health Organization—Eastern Mediterranean Region (WHO-EMR) [16], the Joint United Nations Programme on HIV/AIDS (UNAIDS) [17], and the Global Burden of Disease Study 2015 (GBD 2015) [18]. We are planning to extract data from the included SRs and from the primary studies identified by these SRs from gray literature sources. These SRs and primary studies are likely to be written in English or in the official languages or media of instruction of the selected countries. Hence, as our overview does not apply any language restriction in selecting the SRs and primary studies, we will include in our project those countries having Arabic, English, French, and/or Urdu as primary official languages and/or media of instruction in universities. Arabic, English, French, and Urdu languages are the native languages of the reviewer team.

The 27 identified MENA countries [15–18], their primary official languages [19], media of instruction [20–47], and their selection status in our overview are listed in Table 1. We will include 20 countries, namely Algeria, Bahrain, Djibouti, Egypt, Iraq, Jordan, Kuwait, Lebanon, Libya, Morocco, Oman, Pakistan, Palestine, Qatar, Saudi Arabia, Sudan, Syria, Tunisia, United Arab Emirates, and Yemen. These selected MENA countries have a combined total population of over 560 million people, about 8% of the world’s population [48].

**Table 1:** Countries of the Middle East and North Africa region included and excluded in the overview

Country	The World Bank [15]	WHO-EMR [16]	UNAIDS [17]	GBD 2015 [18]	Official primary languages [19]	Media of instruction in universities and colleges	Inclusion in the overview
Algeria	Yes	No	No	Yes	Arabic	French and Arabic [24]	Yes
Bahrain	Yes	Yes	Yes	Yes	Arabic	Arabic and English [34]	Yes
Djibouti	Yes	Yes	Yes	No	Arabic and French	French [47]	Yes
Egypt	Yes	Yes	Yes	Yes	Arabic	Arabic and English [30]	Yes
Iraq	Yes	Yes	Yes	Yes	Arabic	Arabic and English [29]	Yes
Jordan	Yes	Yes	Yes	Yes	Arabic	Arabic and English [26]	Yes
Kuwait	Yes	Yes	Yes	Yes	Arabic	Arabic and English [42]	Yes
Lebanon	Yes	Yes	Yes	Yes	Arabic	Arabic, French, and English [31]	Yes
Libya	Yes	Yes	Yes	Yes	Arabic	Arabic and English [25]	Yes
Morocco	Yes	Yes	Yes	Yes	Arabic	Arabic and French [27]	Yes
Oman	Yes	Yes	Yes	Yes	Arabic	Arabic and English [35]	Yes
Pakistan	No	Yes	No	No	Urdu	English [39]	Yes
Palestine	Yes	No	Yes	Yes	Arabic	English and Arabic [20]	Yes
Qatar	Yes	Yes	Yes	Yes	Arabic	Arabic and English [43]	Yes
Saudi Arabia	Yes	Yes	Yes	Yes	Arabic	Arabic and English [46]	Yes
Sudan	No	Yes	Yes	Yes	Arabic	English [38]	Yes
Syria	Yes	Yes	Yes	Yes	Arabic	Arabic, French, and English [28]	Yes
Tunisia	Yes	Yes	Yes	Yes	Arabic	Arabic and French [45]	Yes
United Arab Emirates	Yes	Yes	Yes	Yes	Arabic	English [22]	Yes
Yemen	Yes	Yes	Yes	Yes	Arabic	Arabic and English [39, 44]	Yes
Afghanistan	No	Yes	No	Yes	Dari and Pashto	English and Arabic [21]	No
Cyprus	No	Yes	No	No	Greek	Greek, Turkish, and English [33]	No
Israel	Yes	No	No	No	Hebrew	Hebrew and English [41]	No
Iran	Yes	Yes	Yes	Yes	Farsi	English and Farsi [23]	No
Malta	Yes	No	No	No	Maltese	Maltese and English [32]	No
Somalia	No	Yes	Yes	No	Somali	Somali, Arabic, and Italian [36]	No
Turkey	No	No	No	Yes	Turkish	Turkish and English [40]	No

We will include SRs on population health in MENA published since 2008—the publication year of the first version of the Cochrane Handbook for Systematic Reviews of Interventions [4]. We will not restrict our overview to any health condition or intervention. Our primary outcomes are gray literature sources in SRs on population health in MENA and study-level quality in the gray literature. In the current project, we will use the definition of gray literature provided at The Twelfth International Conference on Gray Literature in Prague in 2010 [8]. “Grey literature stands for manifold document types produced on all levels of government, academics, business and industry in print and electronic formats that are protected by intellectual property rights, of sufficient quality to be collected and preserved by libraries and institutional repositories, but not controlled by commercial publishers; i.e. where publishing is not the primary activity of the producing body.”. Additionally, we will consider population health being defined as “the health outcomes of a group of individuals, including the distribution of such outcomes within the group” [49].

In order to provide an exhaustive list of gray literature sources, we will check the reference list of any review on available population health data sources in MENA identified during the multilevel screening process. If we identify relevant citations of gray literature sources related to population health in MENA, we will add them to our provided list of gray literature sources.

### Information sources and search strategy

We will conduct a literature search (by AA) on PubMed [50] (Additional file 2). Key search terms will be related to countries’ names, MENA populations’ names, and MENA sub-regions’ names such as North Africa, East Africa, and the Middle East. We will construct a broad search criteria using Boolean logic (OR and AND) to combine Medical Subject Headings (MeSH) terms and Title/Abstract words. We will use the MeSH Database [50] to search and identify relevant MeSH terms and use filters selecting reviews, SRs, and meta-analyses. The search will be limited to articles published since 2008.

### Data management

All reviews, SRs, and meta-analyses identified on PubMed will be imported into Endnote [51] and duplicates will be removed by HA; checking of this step will be conducted by KC. Two reviewers (AA and HA for publications in English, other combinations of reviewers for the remaining languages) will independently conduct a multilevel screening on Rayyan software, which was presented at the 22nd Cochrane Colloquium [52, 53]. In order to select potentially relevant SRs, we will first screen titles and abstracts of all unique reviews. We will then screen the included full texts to select the relevant SRs that will be included in our overview. Any disagreements during the screening process will be resolved by discussion and consensus between the reviewers. In the Title/Abstract screening step, we agreed that will be inclusive: if one of the reviewers thinks that the report needs to go through a full-text screening, we will include the report in this following step. For the full-text screening step, consensus is defined as > 50% of agreement between the authors. Reasons of exclusion in each screening steps will be recorded. One reviewer (AA for SRs and primary studies published in English, other reviewers according to the languages) will extract data on Statistical Package for the Social Sciences (SPSS), while a second reviewer (KC for SRs and primary studies published in English, other reviewers according to the languages) will check 100% of the extracted data. We will extract SRs’ characteristics such as authors, country, corresponding author’s institution, title, journal, year of publication, literature sources, SR period coverage, SR geographical coverage, and populations included. From the SRs, we will also extract characteristics of primary studies identified from gray literature (references, language and format of the study publication, risk of bias assessment, sampling methodology, health issue status, and response rate). Whenever one of primary studies’ characteristics is not available in the corresponding SR, we will retrieve and extract data from the primary study report, if possible. A study report refers in previous reviews to a publication such as an article, a conference abstract, or a country-level report that presents study outcomes [54, 55]. Any disagreements during the extraction process will be resolved by discussion and consensus between the reviewers (> 50% agreement between the reviewers).

### Assessment of methodological quality of included systematic reviews

Using the Assessment of Multiple Systematic Reviews (AMSTAR) tool, we will assess the methodological quality of included SRs [56, 57].We will use AMSTAR tool rather than its revised version (R-AMSTAR), since R-AMSTAR is not yet validated [58] and since AMSTAR was recommended for assessing observational studies [57, 58]. More specifically, we will appraise included SRs in our overview regarding the method used in literature searching, study selection, data collection, data analysis, publication bias assessment, and conflict of interest statement.

### Data synthesis and evidence mapping

We will summarize SRs’ methodology with a descriptive approach using tables presenting key characteristics of gray literature sources and quality of studies included from gray literature. From the SR-level quality assessment, we will estimate the overall proportion of SRs that used gray literature as one of their data sources. We will also provide a list of gray literature sources identified in the included SRs stratified by country and by research topic. This list format will be similar to the Canadian Agency for Drugs and Technologies in Health checklist “Grey Matters” in the field of drugs and technologies in health [59]. This Canadian list is used to ensure the retrieval of evidence-based agency reports, to help document the gray literature search process, and to conduct the gray literature search in a comprehensive way [59].

In the subgroup analysis, we will assess the differences between SRs that use gray literature and those that do not. We will estimate the proportion of SRs that used gray literature according to whether these SRs were produced by institutions located in MENA or outside MENA. We will compare language and format of gray literature sources included in SRs produced by institutions in MENA and among those produced by institutions outside MENA. Student’s *t* test and chi-squared test [60] will be used to determine whether the differences between subgroups are statistically significant (*p* value < 0.05). Bonferroni correction will be used to address the multiple testing issue regarding false positive.

Additionally, for each MENA country, we will review the five latest SRs containing gray literature searching. From the SRs, we will report on primary study-level quality assessment. We consider that SRs’ authors are the experts in their research topics; as such, we will rely on their study-quality assessment. We are not aiming to compare the study-level quality between SRs but to estimate the overall proportion of good quality studies from the gray literature related to population health using the primary-study quality assessment. This proportion of good-quality primary studies included in our overview will be estimated after excluding duplicate studies using Endnote software [51]. When an SR does not provide quality assessment, we will assess study-level quality of included studies from the gray literature based on the Cochrane Handbook for Systematic Reviews of Interventions [4]. As described in previous reviews, we will classify studies as having a low, high, or unclear risk of bias in each of the three quality domains (sampling methodology, disease ascertainment, and response rate) [54, 55].

We will construct an evidence gap map defined as a visual depiction of the characteristics of evidence in a particular field [61] highlighting the type and quality of the gray literature used in published SRs. This evidence gap map will also emphasize existing gaps in gray literature searching [61]. This map will show the quality and the quantity of studies per language from gray literature included in SRs on population health in MENA. Our evidence gap map can aid in emphasizing gaps in SRs’ literature searching and in planning future SRs on population health in MENA.

### Reporting of the findings

Our overview will be reported following the preferred reporting items for overviews of systematic reviews [62] As overviews have a similar structure as systematic reviews, but include systematic reviews instead of primary studies [4], our overview’s abstract will be reported following the PRISMA for Abstracts Checklist [63] and its methodological quality will be assessed using AMSTAR tool [56, 57]. The findings will be disseminated via publication of a manuscript in a peer-reviewed journal and presented at relevant conferences.

## Discussion

Our overview of SRs will enable us to identify, map, and compare the use of gray literature sources in published SRs on population health in MENA. The findings of this overview of SRs will have implications for research and evidence-based clinical and policy decisions. Our results will provide insight on available gray literature sources related to population health in MENA. The overview will describe to what extent researchers are missing reliable information when conducting SRs without including gray literature or when including a specific type of gray literature source. Our project will inform researchers, clinicians, and policymakers about the importance of including an extensive gray literature search in a region where official languages and/or media of instruction in universities include languages other than English.

### Ethics

Ethical approval will not be needed, as this is a systematic review. Data used will not be individual patient data; therefore, there will be no concerns about privacy.

## Additional files

Additional file 1: PRISMA-P* 2015 checklist: recommended items to address in a systematic review protocol [64]. (DOCX 27 kb)

Additional file 2: Search criteria. (DOCX 18 kb)

## Abbreviations

AMSTAR: Assessment of Multiple Systematic Reviews
GBD 2015: Global Burden of Disease Study 2015
IOM: Institute of Medicine
MENA: Middle East and North Africa
MeSH: Medical Subject Headings
PICO: Population-intervention-comparator-outcome model
PRISMA 2009: Preferred Reporting Items for Systematic Reviews and Meta-Analyses
PRISMA-P 2015: Preferred Reporting Items for Systematic Reviews and Meta-Analyses Protocols
SPSS: Statistical Package for the Social Sciences
SR: Systematic review
UNAIDS: Joint United Nations Programme on HIV/AIDS
WHO-EMR: World Health Organization—Eastern Mediterranean Region
